# The British Journals, &c.: Midwifery, and Diseases of Women and Children

**Published:** 1842-10

**Authors:** 


### MIDWIFERY, AND DISEASES OF WOMEN AND CHILDREN.

Extraordinary Case of Twins. On the 3d of April last, Dr. Jamieson was called to visit a lady thirty years of age, in consequence of severe pain in the abdomen, recurring at uncertain intervals, and lasting generally about five minutes at a time.

The author discovered a firm hard tumour, reaching as high as the umbilicus, which softened on the subsidence of pain, and appeared to be the gravid uterus. On applying the stethoscope, Dr. Jamieson thought he heard a placental murmur in the right iliac fossa; but the lady said it was impossible she could be with child, as she had been confined so recently (seven weeks before), and was at present nursing. As, however, Dr. Jamieson was convinced that the tumour was the uterus, and that it was acting to get rid of something, he ordered a dose of oil and retired to another room, in order to explain to the husband that he believed there was some foreign body in the uterus of his wife.

The author was hurriedly summoned, while engaged in this explanation, to the apartment of the lady: and on examining per vaginam, found the head of a small child presenting, with the membranes entire, which, on the occurrence of another pain, was expelled together with the placenta. The child was dead, and seemed to be about the sixth month of gestation; and though shrivelled and dark, was not at all putrid or decomposed. It was between eight and nine inches long. The mother was of course greatly surprised. She had been confined of the other twin on the 13th of February. Consequently the dead fœtus must have remained in the womb for forty-nine weeks.—Dr. Jamieson, Dublin; *Dublin Journal of Med. Science*, No. lxiv. *Sept*. 1841.

---

Case of Short Funis. The funis in this case was only seven inches and a half. In Dr. Churchill's tables, the average length is eighteen inches; the next most frequent length, twenty-four inches. The shortest cord in 500 cases of the latter gentleman was twelve inches, the longest fifty-four. Mr. Stone, however, has met with a shorter funis than that of the present case, namely one of only six inches.

Dr. Thomson is of opinion that a quick delivery of the fœtus is always or generally followed by a prompt expulsion of the placenta.—Dr. J. B. Thomson, London; *Lancet*, No. x. *June* 4, 1842.

---

Case of Arm Presentation, in which Turning was impracticable. The membranes had given way at 9 o'clock of the preceding night, and Dr. Lynn found the right hand and arm protruding from the vagina, the shoulders and part of the thorax and neck of the child being firmly impacted in the upper part of the pelvis. With every justifiable effort which Dr. Lynn was capable of making, he could not succeed in seizing the feet, but got his forefinger into the ham of the right leg, as it lay bent upon the thigh towards the sacrum of the mother. The author was utterly unable to bring down the leg. He, therefore, now eviscerated the thorax, and with great difficulty succeeded in seizing the right foot, in bringing it down, and in applying a fillet around it. But although he now applied all justifiable traction, he was unable to move the child out of its impacted position. Accordingly, he let out the contents of the abdominal cavity, and with the crotchet brought down the pelvis of the fœtus. The operative part of the proceeding occupied about an hour; not half a pint of blood was lost before or after delivery, This is the third successive unnatural presentation which has occurred to this woman. who, notwithstanding, has made an excellent recovery.—Dr. Lynn ; *Dublin Medical Press*, No. 190, *August* 24, 1842.

---

Case of Uterine Polypus, and of Short Funis. A woman was delivered on the 4th of December, and on the 6th she was again taken, with what she supposed to be labour-pains. Mr. Collyns on examining, found a large substance partly in the vagina and partly in the uterus, which was soon expelled by the efforts of the womb. It proved to be an organized tumour, of an oblong form, which had been attached to the womb by a neck about two inches long, and the thickness of one's finger. The tumour, which was perfectly solid, looking like flesh, with small arteries and veins, running through it, measured sixteen inches in length, eleven in circumference, and weighed two pounds and a quarter. The woman got perfectly well.

The same practitioner mentions another case in which the funis was only, or not quite, six inches long. This case was marked by alarming hemorrhages during pregnancy, but not during parturition.—Wm. Collyns, Esq.; *Prov. Med. Journal*, No. xviii. *August* 6, 1842.

---

Case of Triplets. On examination, the breech was found presenting, with a hand hanging quite out of the passage, showing that it was a case of twins. The feet were easily reached, and the child, a female, was delivered. During the whole of this time, the arm remained hanging out, but receded as soon as the first child was born; and the right shoulder presenting, the mother was, after sometime, delivered of a dead male child, connected with the cord of which was a double placenta. Fresh pains came on, and a third child, a male, came away. The third child was in no way connected with the first two.

The woman had attained her full time, and three children were all perfectly formed; weighed six pounds and a half each, and were sixteen inches long.—Dr. David, Edinburgh; *London and Edin. Jour. of Med. Science*, No. vii. *July*, 1842.

---

Large Congenital Tumour. A male child was born on the 22d of July last, with a tumour extending from the middle of the *os frontis* to within about an inch of the ear, and from the upper eyelash to the coronal suture. Its circumference round the base was nine inches; its substance was firm and doughy; its shape conical, on the apex it contained fluid. Round its base, the bone was raised into a circular ridge, but an opening into the brain could not be felt. The child is in good health and thriving. *The mother states that, in an early period of her pregnancy, she had been disgusted by the sight of the entrails of a pig.*—Alex. Bredon, Esq.; *Dublin Medical Press*, No. 188. *August* 10, 1842. [In our foreign selections of this number, a remarkable case is given of the impressions which may be made on the fœtus through the mother.]

